# Bilateral brachial plexus injury following acute carbon monoxide poisoning

**DOI:** 10.1186/2050-6511-14-61

**Published:** 2013-12-07

**Authors:** Mounia Rahmani, Halima Belaidi, Maria Benabdeljlil, Wafa Bouchhab, Nadia El Jazouli, Asmae El Brini, Saadia Aidi, Reda M Ouazzani, Mustapha El Alaoui Faris

**Affiliations:** 1 Department of Neurology A and Neuropsychology, Hôpital des spécialités, Ibn Sina University Hospital, University Mohamed V Souissi, Avenue abderrahim bouabid, Rabat 10100, Morocco; 2 Department of Neurophysiology, Hôpital des spécialités, Ibn Sina University Hospital, University Mohamed V Souissi, Rabat, Morocco

**Keywords:** Peripheral neuropathy, Carbon monoxide, Volkmann’s contracture, Brachial plexus

## Abstract

**Background:**

Carbon monoxide (CO) intoxication is a leading cause of severe neuropsychological impairments. Peripheral nerve injury has rarely been reported. It consists usually in a demyelinating polyneuropathy or mononeuropathy affecting mainly the lower limbs. Isolated involvement of both upper extremities has been described in only 4 patients related to root damage. We report the first case of bilateral brachial plexus injury following CO poisoning and review all previous CO-induced neuropathy described in literature.

**Case presentation:**

After being unconscious for three hours, a 42 years old man experienced bilateral brachial weakness associated with edema of the face and the upper limbs. Neurological examination showed a brachial diplegia, distal vibratory, thermic and algic hypoesthesia, deep tendon areflexia in upper limbs. There was no sensory or motor deficit in lower extremities. No cognitive disturbances were detected. Creatine kinase was elevated. Electroneuromyogram patterns were compatible with the diagnosis of bilateral C5 D1 brachial axonal plexus injury predominant on the left side. Clinical course after hyperbaric oxygen therapy was marked by a complete recovery of neurological disorders.

**Conclusion:**

Peripheral neuropathy is an unusual complication of CO intoxication. Bilateral brachial plexus impairment is exceptional. Various mechanisms have been implicated including nerve compression secondary to rhabdomyolysis, nerve ischemia due to hypoxia and direct nerve toxicity of carbon monoxide. Prognosis is commonly excellent without any sequelae.

## Background

Carbon monoxide (CO) intoxication is the most common lethal intoxication all over the world. It affects multiple organs such as brain, kidney, heart, lung, skeletal muscle and peripheral nerve. Most complications have been widely described, but peripheral neuropathy and much more electrophysiological features have scarcely been reported [1]. We present a case of reversible bilateral brachial plexus damage following CO poisoning with electrophysiological analysis.

## Case presentation

A 42-year-old man, suffered in the 31th of January 2011 from an accidental CO poisoning due to a failed water heater used in the bathroom. After being unconscious for three hours, he woke up with brachial weakness associated with edema of the face and the upper limbs. He received normobaric oxygen therapy during 30 minutes. On admission three days later, neurological examination showed a brachial diplegia (all muscles were equally affected with motor strength evaluated at 1/5), distal vibratory, thermic and algic hypoesthesia, deep tendon areflexia in upper limbs. There was no sensory nor motor deficit in lower extremities. No cognitive disturbances were detected. Biological tests showed elevated creatine kinase at 693 UI/L, normal glucose, electrolytes, hepatic function and complete blood count tests. Cerebral magnetic resonance imaging (MRI) showed bilateral pallidal abnormalities. Cervical spine MRI was normal. Electroneuromyography (ENMG) performed 9 days after intoxication showed minor signs of bilateral C5 D1 brachial plexus injury. It was then repeated 2 weeks later. Motor nerve conduction was normal except for the left median motor amplitude which was highly decreased (Table 1). Sensitive amplitudes for the left median and radial nerves were reduced and absent for both cubital nerves (Table 2). Needle electromyography revealed bilateral reduced recruitment pattern in different upper muscles (deltoid, biceps brachii, supinator longus, triceps brachii, radialis muscles, the first dorsal interosseous muscle, abductor pollicis brevis and adductor pollicis muscle) without denervation potentials such as fibrillations/positive waves. ENMG pattern were compatible with the diagnosis of bilateral C5 D1 brachial axonal plexus injury predominant on the left side. Patient underwent 10 sessions of hyperbaric oxygen therapy. Evolution was marked by the remission of sensory and motor deficits as well as the regression of the edema. After 4 months, patient remained free of any sequelae.

**Table 1:** Electromyography with results of motor conduction study

**Nerves**	**DML (ms)**	**Distal CMAP amplitude (mV)**	**Proximal CMA**	**Motor conduction velocity (m/s)**	**F wave (ms)**
**Muscles recorded**	**Referential values**	**Referential values**	**P amplitude (mV) values**	**Referential values**	**Referential values**
**Right median**	3.1	4.3	3.6	51.1	25.4
**(APB)**	<4	> 5	> 5	> 48	<31
**Right ulnar**	2.8	8	6.6	52.6	25.8
**(APM)**	<3.8	>.5	> 5	> 47	<31
**Left median**	3.1	0.7	0.5	62.9	32.4
**(APB)**	<4	> 5	> 5	> 48	<31
**Left ulnar**	2.6	6.3	4.6	63.8	31.2
**(APM)**	<3.8	> 5	> 5	> 47	<31

APB: abductor pollicis brevis, APM: adductor pollicis muscle, DML: Distal motor latency, CMAP: compound muscle action potential.

**Table 2:** Electromyography with results of sensory conduction study

**Nerves**	**Latency (ms)**	**SNAP amplitude (μV)**	**Sensitive conduction velocity (m/s)**
**Muscles recorded**	**Referential values**	**Referential values**	**Referential values**
**Right median (finger 3- wrist)**	3.9	6.9	37.2
	<3	>10	>40
**Right ulnar (finger 5- wrist)**	0	0	0
	<2,4	>7	>40
**Left median (finger 3- wrist)**	3.3	3.9	44.6
	<3	>10	>40
**Left ulnar (finger 5- wrist)**	0	0	0
	<2,4	>7	>40
**Right radial**	7.9	11.6	17.5
	<2	>11	>41
**Left radial**	8.9	4.3	17.9
	<2	>11	>41

Sensory nerve action potential: SNAP.

## Discussion

Central nervous system manifestations of CO intoxication are well known, but little has been written regarding peripheral neuropathies. Claude was the first author who reported diffuse nerve demyelination in 2 patients [2]. Few cases of CO-induced neuropathies have been described later: isolated radial paralysis, median and facial nerves paralysis, brachial plexus injury secondary to axillar hematoma, sciatic paralysis due to nerve hematoma [3-5].

To our knowledge, 8 reports of neuropathy after CO intoxication have been published last decades [6-13]. Only one series has described CO induced neuropathy [11,12]. In this study, of 2759 patients admitted to hospital for CO poisoning and examined clinically between 1976 and 1982, neuropathy was diagnosed in 23 subjects. The incidence of neuropathy was 0,8% in all admitted cases. There were 11 men and 12 women with ages ranging from 17 to 52 years old. Among all patients, 14 had sensory symptoms, 8 mixed symptoms and only one exhibited isolated motor symptoms. All cases except two involved the lower extremity. There was one case of facial paralysis. Local swelling of the affected side was found in 20 patients and acute renal failure developed in 7 others. On ENMG, all patients presented mild to moderate denervation potentials while nerve conductions velocities were normal in seven. An asymptomatic extremity was affected in four patients, as suggested by ENMG study. Evidence of lumbosacral radiculopathy was noted in five patients. There were 12 cases of mononeuropathy and 9 of polyneuropathy. In 2 cases, the type of neuropathy was not precised. In two patients, pathologic findings of sural nerve and muscle biopsies were consistent with demyelination and muscle necrosis. The author concluded that from his experience, neuropathy after CO poisoning affects usually young adults of both sexes, is exclusively confined to the lower extremity, has motor and sensory symptoms, occurs in nerve root as well as peripheral nerve, is promoted by localized swelling secondary to rhabdomyolysis and complete recovers after 3 to 6 months [11,12].

Other articles have reported sporadic peripheral neuropathy: polyradiculonevritis [7], polyneuritis [9,14], isolated bilateral facial paralysis [8], optic neuropathy [10], phrenic nerve paralysis [13] and Volkmann’s contracture [6,11].

More recently, the only detailed electrophysiological study of CO induced neuropathy has been published [1]. ENMG study in this case showed abnormalities compatible with mixed sensory and motor neuropathy in both symptomatic (right) and asymptomatic (left) lower limb. The absence of denervation potentials in right weak tibialis anterior muscle suggested that axonal damage was not the main pathogenic mechanism involved. Patient has completely recovered and electrophysiological study performed 1 year later returned to normal.

Our patient experienced reversible bilateral brachial plexus injury following acute CO poisoning with total improvement after few months. It represents the third case published in literature with isolated involvement of bilateral upper limbs. Choi mentioned one case of bilateral Volkmann’s contracture in his series and Snyder reported a bilateral symmetrical ulnar paralysis in a 25 years old man 4 days after recovering from coma [8,11]. Paralysis in our patient was caused by bilateral brachial plexus injury. There is no publication until now reporting bilateral involvement of brachial plexus after CO poisoning.

Electrophysiological study after CO intoxication reveals findings compatible with a motor and sensory peripheral neuropathy with mostly radicular damages [11]. Nerve biopsy shows usually demyelination with preservation of axons explaining the excellent prognosis with total improvement often observed [2,7,15]. However, Choi in his series hypothesized a concomitant axonal involvement. This nonspecific finding offers little help in determining pathogenesis of CO neuropathy. At least 4 different mechanisms may be responsible: ischemia due to hypoxia induced by CO; petechial hemorrhages; cytotoxic effect of CO itself and direct nerve compression or venous obstruction with ensuing edema and circulatory compromise in an unconscious patient. This last mechanism resulting in a compartment syndrome is usually evoked in case of mononeuropathy or Volkmann’s syndrome. However, interruption of the arterial blood flow due to mechanical pressure while the patient is unconscious for several hours and the increased subfascial edema due to muscle necrosis appear to combine with the already anoxic state produced by CO. The appearance of symmetrical peripheral nerve lesions in our patient is against compressive or petechial hemorrhage mechanisms. Most likely neuropathy resulted from combination of ischemic and toxic factors. Improvement of edema may have contributed to the recovery of the neuropathy.

## Conclusion

Central nervous system complications following CO poisoning are well reported in the literature but peripheral neuropathy is under-recognized. The severity of the central symptomatology tends to overshadow involvement of the peripheral nervous system. CO induced neuropathy is commonly a motor and sensory demyelinating mononeuropathy or polyneuropathy of the lower extremities and occurs usually in young adults. It is often associated with local swelling caused by muscle necrosis, which may be an important contributing factor to the development of the neuropathy. The pathologic finding in most reported cases is demyelinating and the prognosis is excellent. The originality of our case is the isolated involvement of the upper limbs exceptionally reported, and the presence of bilateral plexus injury, never described until now.

### Consent statement

Informed consent was obtained from the patient for publication of this Case report. A copy of the written consent is available for review by the Editor of this journal.

## Competing interest

We declare Professors Rahmani Mounia, Belaidi Halima, Benabdeljlil Maria, Aidi Saadia, Ouazzani Mohamed Reda, El Alaoui Faris Mustapha and Doctors Bouchhab Wafa, El Jazouli Nadia, El Brini Asmae have no competing interests.

## Authors’ contributions

RM: FG, -acquisition, analysis and interpretation of data, -literature research, -analysis of articles concerning similar publications, -conception and drafting the article. BH: FG, -electrophysiological study, -analysis of data from electromyogram. BW, EJN, EBA: FG, -acquisition of electrophysiological results, -analysis of data. OMR: FG, -analysis of data from electromyogram. BM, AS, EAFM: FG, -interpretation of data. All authors read and approved the final manuscript.

## Pre-publication history

The pre-publication history for this paper can be accessed here:

http://www.biomedcentral.com/2050-6511/14/61/prepub
